# Clinical Features and Outcomes of Patients With COVID-19 Infection and Acute Kidney Injury Requiring Hemodialysis in an Intensive Care Unit: A Retrospective Study From a Tertiary Care Center in Eastern India

**DOI:** 10.7759/cureus.75363

**Published:** 2024-12-09

**Authors:** Harsh Vardhan, Megha Saigal, Shyama Shyama, Amresh Krishna

**Affiliations:** 1 Nephrology, Patna Medical College, Patna, IND; 2 Nephrology, All India Institute of Medical Sciences, Patna, Patna, IND; 3 Internal Medicine, All India Institute of Medical Sciences, Patna, Patna, IND

**Keywords:** acute kidney injury, covid-19, hemodialysis, intensive care, mortality, risk factor

## Abstract

Background: During the COVID-19 pandemic, it has been observed that acute kidney injury (AKI) especially requiring intervention support of hemodialysis has notably increased mortality rates among COVID-19-positive critically ill patients; however, comprehensive data regarding this from India, especially the eastern territory, remains sparse. This study aims to outline the demographic, clinical, and biochemical characteristics, along with the outcomes, of these patients.

Methods: A retrospective study was performed at the All India Institute of Medical Sciences (AIIMS), Patna, from March 1, 2020, to March 31, 2021. Included were patients diagnosed with COVID-19 and AKI necessitating hemodialysis during their intensive care unit (ICU) stay. These patients tested positive for COVID-19 and met the Kidney Disease: Improving Global Outcomes (KDIGO) criteria for AKI stages 1-3, requiring ICU admission and hemodialysis. Medical history, clinical features, laboratory results, comorbidities, and demographic data were collected and analyzed. Patients were tracked from admission to discharge or death. Gaussian-distributed values were compared using the unpaired t-test or Pearson's test, while non-Gaussian continuous variables were analyzed using the Mann-Whitney test or Spearman's test. The study employed the Kolmogorov-Smirnov test to assess Gaussian distribution, while categorical data were compared using the Chi-square test.

Results: Among 773 patients with positive COVID-19 tests who were admitted to the ICU, 236 patients developed AKI, and among them, 139 patients required hemodialysis. The total mortality rate was 167 (70.7%) among people who had AKI and 102 (77%) in patients with AKI who required hemodialysis. AKI was also a risk factor associated with higher mortality rates in older patients (>45 years) (n=150 (73.2%)), those needing invasive ventilation (n=163 (88.1%)), and patients with elevated total leucocyte count (TLC) (n=130 (79.3%)), lactate dehydrogenase (LDH) (n=159 (72.9%)), interleukin-6 (IL-6) (n=153 (72.2%)), and serum ferritin (n=51 (73.7%)) and hypoalbuminemia (n=152 (73.1%)).

Conclusion: AKI requiring hemodialysis significantly increases mortality risk in COVID-19 patients. Other risk factors for mortality with AKI in COVID-19-positive patients include age, elevated leucocyte count, invasive ventilation, and deranged inflammatory markers.

## Introduction

A new virus known as SARS-CoV-2 surfaced in Wuhan, China, in December 2019. Millions of people have died from it globally, and symptoms frequently progress from pneumonia to acute respiratory distress syndrome (ARDS). The COVID-19 outbreak was declared a pandemic by the World Health Organization in March 2020, with potentially disastrous outcomes [1]. COVID-19 infection is primarily a respiratory disorder, but with an increase in severity, it involves other major organs such as the heart and kidneys [2-4]. Nonetheless, prior research has also documented the involvement of several organs, such as the kidney, liver, and heart. In fact, a notably high percentage of SARS-CoV-2 patients have been documented to have acute kidney injury (AKI), especially in those with serious medical conditions [5]. There are large variations in the literature about the reported incidence of AKI. Estimates for COVID-19-infected patients range from 6.5% to 46% [6,7], with serious cases having the largest range (23%-81%) [8,9]. Information about AKI in patients with severe COVID-19 is scarce [10]. AKI is a frequent side effect of severe COVID-19 infections that affects 33%-43% of hospitalized patients [11,12].

There is little understanding of the AKI mechanism in COVID-19 patients. The proposed mechanisms include ischemic tubular necrosis [12-14], hemodynamic instability, direct viral infiltration of podocytes and renal proximal tubular cells, cytokine storm, mechanical ventilation, and the use of nephrotoxic drugs [14-16]. Renal endothelial injury affected by SARS-CoV-2 is similar to lung endothelium involvement [17-19]. In a selected subgroup, the diagnosis of COVID-19 was independently determined to be the only cause of the onset of AKI.

There is a lack of data on the clinical and laboratory findings of AKI requiring hemodialysis and their outcomes in patients suffering from COVID-19 from eastern India. Hence, in this study, we have attempted to answer and analyze the baseline characteristics of COVID-19-positive patients developing renal replacement requiring AKI. Additionally, our goal is to find out the risk factor for the patient's mortality, thus providing us with guidance for early recognition and intervention in such cases in the future.

## Materials and methods

Study design

This was an observational retrospective analytical study.

Study site

This study was conducted in a tertiary care center in eastern India.

Duration of study

This retrospective study was performed from March 1, 2020, to March 31, 2021.

Inclusion criteria

All patients diagnosed with documented COVID-19 infection and AKI who required hemodialysis during ICU stay were included as study participants, as well as patients who developed AKI requiring hemodialysis.

Exclusion criteria

We excluded patients less than 18 years of age and pregnant women.

Statistical test

The unpaired t-test or Pearson's test compared values with a Gaussian distribution. The Mann-Whitney (Wilcoxon rank) test or Spearman's test was applied for continuous variables that did not follow a normal distribution. Gaussian distribution was assessed using the Kolmogorov-Smirnov test, and categorical data were compared using the Chi-square test. Significance was determined with two-tailed p-values set at p < 0.05.

The main goal of the study is to evaluate the demographic profile, clinical findings, and outcomes of these patients. Severe COVID-19 outcomes are defined per the 2020 Centers for Disease Control and Prevention (CDC) guidelines as hospitalization, admission to the intensive care unit (ICU), intubation or mechanical ventilation, or death.

In total, 236 patients fulfilled the inclusion criteria (RT-PCR-positive COVID-19 patients with AKI stages 1-3 as per the Kidney Disease: Improving Global Outcomes (KDIGO) definition [10] and required admission in the intensive care unit). Their data was collected and analyzed. From the time of admission to their eventual discharge or death, the individuals were monitored. Clinical characteristics, laboratory parameters, including inflammatory markers such as ferritin and interleukin-6 (IL-6), and demographic information were recorded, along with medical history and comorbidities.

## Results

During the study period, from the 4,563 inpatients who tested positive for COVID-19 via RT-PCR, 773 had symptoms that required admission to the intensive care unit, and 236 were diagnosed with acute kidney injury based on the KDIGO criteria. Table 1 shows the baseline characteristics of these patients.

**Table 1:** Baseline characteristics of patients at presentation (N=236) *Significant p-value ^Pearson Chi-square test ^#^Fisher's exact test SOB: shortness of breath, COPD: chronic obstructive pulmonary disease

Characteristics	Discharge (n=71)	Death (n=165)	Odds ratio (confidence interval)	p-value
Age (years), number (%)^				
<45 years	16 (51.6)	15 (48.4)	3 (1.3-6.3)	0.005*
≥45 years	55 (26.8)	150 (73.2)		
Gender, number (%)^, male:female				
Male	55 (30.6), 3.4:1	125 (69.4), 3.1:1		
Female	16 (28.6)	40 (71.4)	1 (0.5-1.7)	0.78
Fever^	36 (29.03)	88 (70.97)	1.11 (0.65-1.94)	0.711
Cough^	36 (26.4)	100 (73.5)	1.49 (0.85-2.62)	0.158
SOB^	40 (27.03)	108 (72.97)	1.46 (0.82-2.6)	0.15
Comorbidities				
Diabetes mellitus^	46 (28.2%)	117 (71.8%)	1.3 (0.7-2.4)	0.35
Hypertension^	50 (28.4%)	126 (71.6%)	1.3 (0.7-2.5)	0.34
COPD^#^	5 (31.2%)	11 (68.8%)	0.94 (0.3-3)	0.92
Coronary artery disease^	20 (31.2%)	44 (68.8%)	0.93 (0.5-2)	0.81

The total mortality seen among COVID-19-positive critically ill patients with AKI stage 3 requiring hemodialysis was 102/139 (77%). A significantly higher mortality rate was observed among older patients (more than 45 years) with p-value of <0.05. The discharge ratio was 3.4:1 (male:female), and the mortality ratio was 3.1:1 (male:female). This may be secondary to the absolute number of men being admitted three times more than women (180:56 (male:female)). Those with comorbidities (diabetes mellitus, hypertension, coronary artery disease (CAD), and chronic obstructive pulmonary disease (COPD)) showed a higher mortality; however, it was not statistically significant. The symptoms, such as shortness of breath, cough, and fever, did not show any significant association with the death or discharge of patients.

Table 2 shows the outcomes of patients during the course of treatment. Patients requiring hemodialysis had a higher mortality (77.3%). Also, any form of ventilation (invasive and non-invasive) was found to be a significant predictor of mortality. This reflects the severity of AKI associated with organ dysfunction.

**Table 2:** Characteristics of patients during the course of treatment (N=236) *Significant p-value ^Pearson's chi-square test ^#^Fisher's exact test

Characteristics	Discharge	Death	Odds ratio (confidence interval)	p-value
Dialysis, number (%)^	30 (22.7%)	102 (77.3%)	2.2 (1.3-4)	0.005*
Non-invasive ventilation, number (%)^#^	31 (91.2%)	3 (8.8%)	0.02 (0.007-0.08)	<0.001*
Invasive ventilation, number (%)^	22 (11.9%)	163 (88.1%)	182 (41.2-799.2)	<0.001*

Table 3 shows baseline laboratory parameters when patients were admitted to the hospital. A high mortality rate was noted among those patients who presented with deranged total leucocyte count (TLC) (n=130 (79.3%)), had low serum albumin at the time of admission (n=152 (73.1%)), and had high lactate dehydrogenase (LDH), serum ferritin, and IL-6. There is a significant association with deranged TLC (p<0.001), decreased albumin (p<0.004), deranged LDH (p<0.001), deranged serum ferritin (p=0.001), and IL-6 (p=0.03) with higher morality. Other markers, such as high C-reactive protein (CRP), procalcitonin, D-dimer, lymphopenia, and thrombocytopenia, were not significantly associated with high mortality rates.

**Table 3:** Baseline laboratory parameter of patients with respect to outcome (N=236) *Significant p-value ^Pearson's chi-square test ^#^Fisher's exact test TLC: total leucocyte count, LDH: lactate dehydrogenase, CRP: C-reactive protein

Characteristics (number, %)		Discharge	Death	Odds ratio (confidence interval)	p-value
TLC^	Deranged	34 (20.7%)	130 (79.3%)	4 (2.2-7.3)	<0.001*
					Normal	37 (51.4%)	35 (48.6%)		
	Lymphocyte^	Decreased	64 (29%)	157 (71%)	2.1 (0.1-6.1)	0.15
Normal						7 (46.7%)	8 (53.3%)		
Platelet count^		Decreased	31 (27.9%)	80 (72.1%)	1.2 (0.7-2.1)	0.5
	Normal					40 (32%)	85 (68%)		
	Albumin^	Decreased	56 (26.9%)	152 (73.1%)	3.1 (1.4-7)	0.004*
Normal						15 (53.6%)	13 (46.4%)		
LDH^		Deranged	59 (27.1%)	159 (72.9%)	5.4 (2-15)	<0.001*
	Normal					12 (66.7%)	6 (33.3%)		
	CRP^#^	Deranged	70 (30.3%)	161 (69.7%)	0.6 (0.06-5.2)	0.62
Normal						1 (20%)	4 (80%)		
D-dimer^#^		Deranged	69 (29.7%)	163 (70.3%)	2.4 (0.3-17.1)	0.38
	Normal					2 (50%)	2 (50%)		
	Procalcitonin^	Deranged	45 (28.5%)	113 (71.5%)	1.3 (0.7-2.2)	0.44
Normal						26 (33.3%)	52 (66.7%)		
Serum ferritin^		Deranged	54 (26.3%)	151 (73.7%)	3.4 (1.5-7.3)	0.001*
	Normal					17 (54.8%)	14 (45.2%)		
	Interleukin-6^	Deranged	59 (27.8%)	153 (72.2%)	2.6 (1.1-6.1)	0.03*
Normal						12 (50%)	12 (50%)		
Creatinine^		Deranged	49 (27.5%)	129 (72.5%)	1.6 (0.9-3)	0.13
	Normal					22 (37.9%)	36 (62.1%)		

The risk factors independently associated with mortality are shown in Table 4. Significant among these are invasive mechanical ventilation, deranged total leucocyte count, increased serum ferritin levels, and the severity of COVID-19 infection. With a p-value of 0.007, the adjusted odds ratio of 5.21 (95% CI: 1.55-17.46) indicates a significant correlation between the severity of COVID-19 and the risk of developing AKI. This finding suggests that people who have more severe COVID-19 infections have a much increased chance of experiencing AKI.

**Table 4:** Independent risk factors associated with inhospital mortality *Significant AOR: adjusted odds ratio, TLC: total leucocyte count, LDH: lactate dehydrogenase, CRP: C-reactive protein, IL-6: interleukin-6, COVID-19: coronavirus disease 2019

Characteristics	Adjusted odds ratio (95% CI)	p-value
Age category	2.13 (0.54-8.46)	0.279
Invasive ventilation	286.85 (54.22-1517.4)	0.001*
TLC	3.75 (1.25-11.2)	0.018*
Albumin	2.06 (0.56-7.61)	0.276
LDH	4.32 (0.93-19.9)	0.061
Serum ferritin	5.58 (1.55-19.9)	0.008*
IL-6	1.97 (0.45-8.43)	0.361
Severity of COVID-19 infection	5.21 (1.55-17.46)	0.007*

## Discussion

Our study is a retrospective single-center study conducted by us to look at the incidence, patient demographics, risk factors, and outcomes of AKI among COVID-19 patients receiving hemodialysis and admitted to the intensive care unit of a tertiary care center. It is the first study from eastern India to focus on the association of mortality in AKI patients with severe COVID-19, with severity defined as admission to hospital, need for intensive care, and ventilation requirement (both invasive and non-invasive). A total of 773 severe COVID-19-positive patients were admitted to the ICU of All India Institute of Medical Sciences (AIIMS), Patna. Nearly 30% (n=236) of these patients developed AKI. Nearly 55% (n=132) of patients received hemodialysis. Of the patients who required hemodialysis, 44% (n=102) died during hospitalization. Only 22% (n=30) of patients were discharged after successful treatment.

These data indicate that ICU patients with AKI requiring hemodialysis had a higher mortality rate. The frequency of AKI stages 1-3 ranged from 50% to 81% among the COVID-19 patients in the ICU, according to the KDIGO criteria [20]. Differences in COVID-19 patient severity levels and the diverse methods for estimating missing baseline creatinine may explain the disparities in reported severe AKI rates, leading to up to a 15% difference in AKI incidence. According to the retrospective analysis by Joseph et al. of 100 COVID-19 ICU patients, 37% of them had AKI (stages 2 and 3) [21]. Similar to our study, many global studies with the same cohort of patients found a similar rate of AKI and mortality among hemodialysis-requiring COVID-19-positive patients admitted to the ICU, as shown in Table 5.

**Table 5:** Comparison of our study with similar studies COVID-19: coronavirus disease 2019, ICU: intensive care unit, AKI: acute kidney injury

Study	Number of patients with COVID-19 in the ICU	Incidence of AKI	Dialysis	Outcome (mortality in those with AKI requiring hemodialysis)
Our study	773	236 (30%)	132 (55%)	102/132 (77%)
Mohamed et al. [18]	557	161 (29%)	89 (55%)	64/89 (72%)
Hirsch et al. [12]	9,657	3,854 (39.9%)	638 (6.6%)	505/638 (79.3%)
Gupta et al. [9]	3,099	1,685 (54.3%)	637 (20.5%)	403/637 (63.2%)
Fominskiy et al. [19]	99	72 (75%)	17 (17.7%)	9/17 (52.9%)

We found that patients with ages more than 45 years who were receiving hemodialysis (n=132) had higher mortality (n=102 (43%)). Mortality was also high among patients with pre-existing comorbidities such as diabetes and hypertension. A study from Greece showed that non-survivors were older, had higher Acute Physiology and Chronic Health Evaluation (APACHE II) scores, had a shorter duration of ICU stay, had higher Sequential Organ Failure Assessment (SOFA) scores on admission, and had higher levels of laboratory markers, including creatinine level, ferritin, urea, D-dimer, troponin (TPI), lactate dehydrogenase (LDH), and white blood cells (WBC) [22].

In our study, those AKI patients who received hemodialysis had a mortality rate of 102 (77.3%); both non-invasive and invasive modes of ventilation had high rates of mortality (n=3 (8.8%) and n=163 (88.1%), respectively). The higher rate of mortality among the invasive mode of ventilation is probably due to the maximum number of patients who were initially put on the non-invasive mode of ventilation and lastly put on the invasion mode of ventilation due to worsening of disease. When analyzing the biochemical parameters, it was seen that patients who presented with high total leucocyte counts had higher mortality (n=130 (79%)), as well as those with lymphocytopenia (n=157 (71%)) and thrombocytopenia (n=80 (72%)). Very significantly, decreased serum albumin (n=152 (73%)), high LDH (n=159 (72%)), serum ferritin (n=151 (73.3%)), high interleukin-6 (n=153 (72.2%)), and high values of serum creatinine (n=129 (72.5%)) were also associated with higher mortality. Nicholson et al. in their study analyzed the factors influencing the need for mechanical ventilation and the likelihood of inhospital mortality in COVID-19 patients upon admission. They examined comorbidities, vital signs, and initial laboratory results. The study identified key risk factors, including gender, older age, diabetes mellitus, coronary artery disease, body mass index, SpO2/FiO2 ratio, chronic statin use, and elevated levels of inflammatory and infection markers such as platelet count, neutrophil/lymphocyte ratio, C-reactive protein, procalcitonin, and lactate dehydrogenase. Tailored risk assessments for academic and healthcare audiences using these factors have been created [23].

The extent of cytokine release is directly linked to the risk and severity of AKI, as demonstrated by elevated baseline levels of inflammatory markers such as CRP, LDH, ferritin, and procalcitonin in COVID-19-associated AKI patients, with even higher levels in those requiring renal replacement therapy (RRT) [24]. This observation is consistent with previous research correlating AKI severity to systemic inflammatory response syndrome [25]. Since mortality has also been associated with higher inflammation, these inflammatory parameters may be validated in future studies as an early indicator of these patients having a higher chance of developing AKI requiring RRT, needing ventilator support, and having a higher risk of mortality. Although this study is noteworthy for its large patient number and comparison with existing Indian literature, its observational nature and single-center design cast doubt on the external validity of the findings.

## Conclusions

Acute kidney injury stage 3 requiring renal replacement therapy in the form of hemodialysis in COVID-19 patients who are admitted to the intensive care unit is associated with a significant increase in mortality risk. Other risk factors for increased mortality seen in patients with AKI include older age group, elevated leucocyte count, invasive ventilation, and deranged inflammatory markers such as serum ferritin and interleukin-6.
